# Effect of Rare Earth Elements on the Morphology of Eutectic Carbides in AISI D2 Tool Steels: Experimental and Modelling Approaches

**DOI:** 10.1038/s41598-018-27658-w

**Published:** 2018-06-18

**Authors:** Klemen Zelič, Jaka Burja, Paul John McGuiness, Matjaž Godec

**Affiliations:** 1 Faculty of Mechanical Engineering, University of Ljubljana, Laboratory for internal combustion engines and electro mobility, Ljubljana, SI-1000 Slovenia; 2 Institut of Metals and Technology, Metallic Materials and Technology Department, Ljubljana, SI-1000 Slovenia; 3 Institut of Metals and Technology, Physics and Chemistry of Materials Department, Ljubljana, SI-1000 Slovenia; 4 Jozef Stefan International Postgraduate School, Nano Science and Nano Technology, Ljubljana, SI-1000 Slovenia

## Abstract

The morphology of the eutectic chromium carbides in the microstructure of as-cast AISI D2 tool steel was modified by adding small amounts of rare-earth elements (REEs) to the melt. As a result of these REE additions the eutectic carbide morphological type was changed from lamellar to globular. Similar phenomena have already been reported for various tool steels, but no complete theoretical explanation has been provided. Here, we propose a new model that is derived from first-principles thermodynamic calculations based on the phase-field modeling of the eutectic reaction. Using this new approach, where the decomposition of the phase-boundary surface-energy term is divided into the isotropic and anisotropic parts, we were able to account for the transition from a lamellar to a globular eutectic morphology in REE-modified AISI D2 tool steel.

## Introduction

AISI D2 steel from the group of cold-work tool steels is used when wear resistance is important, such as blanking, forming dies and thread-rolling dies, cutting tools, stamping, woodworking and molding tools for plastics^1^. Due to the high carbon and chromium contents, large eutectic chromium carbides form during the solidification. Carbides in the as-cast microstructure are undesirable because they are points where cracks initiate during the steel’s post processing (usually forging). One solution to this problem is to use powder metallurgy, which gives us good control over the microstructure’s evolution. But due to the high cost of powder metallurgy, casting is often the only realistic option. Thus, it is important to investigate ways in which we can control the process of casting and solidification, for example, by using small amounts of modifying agents (different chemical elements) that are added to the molten steel in order to influence the microstructure during solidification.

In the case of high-chromium, high-carbon tool steel several attempts to modify the morphology, size, volume, distribution and type of eutectic carbides with various modifying agents are reported. Pan *et al*.^2^ showed that by adding a K/Na modifier to high-speed steels, the distribution and morphology of the eutectic carbides can be changed. A modifier can affect the initial number of nuclei and consequently refine the microstructure^2–4^. On the other hand, adding niobium and titanium refined the eutectic chromium carbides in high-chromium cast iron (HCCI) and high-alloy tool steels. The presence of these elements can lead to the formation of TiC and NbC carbides at a higher temperature than the eutectic carbides, so reducing the carbon content in the melt and thereby the volume fraction of the eutectic carbides^5,6^. It was also confirmed that the addition of boron could alter the carbide morphology of high-chromium ledeburite steel from a strip eutectic and rosette eutectic into a divorced/phase-separated eutectic. Moreover, they studied the effect of trace additions of a REE–Te–B (REE = rare-earth element) multiple modifier on the microstructure of CD-2 steel and concluded that with the combined addition of trace amounts the eutectic carbide morphology can be changed^3,7^.

In analogy with nodular cast iron where a morphological modification of graphite is achieved by adding magnesium and cerium to gray cast iron^8^, we decided to modify AISI D2 tool steel by adding REEs. These elements have been shown to improve high-alloy tool steels and have the ability to change the morphology, type and distribution of eutectic carbides in the as-cast microstructure. Many experiments have been made on AISI M2 high-speed tool steel^3,9,10^. It was reported several times, for example, that a morphological change of the eutectic carbides can be observed in REE-modified M2 tool steel^3,11–13^. Gao *et al*.^14^ investigated AISI H13 tool steel modified with REEs and reported a change in the morphology and the type of eutectic carbides in the as-cast microstructure as well as improved mechanical properties of the steel. Hamidzadeh *et al*.^15^ reported a dramatic change in the eutectic carbide’s morphology due to a REE modification in AISI D2 tool steel with a chemical composition almost identical to ours. Using four different REEs, the modified samples showed a change in the morphology of the eutectic *Cr*_7_*C*_3_ carbides from a lamellar to a globular eutectic.

The most popular theoretical explanation for the modification is based on the effect of modified non-metallic inclusions in the melt acting as heterogeneous nuclei for the nucleation of the eutectic carbide^14,15^. We find such an explanation highly unlikely due to the fact that after a detailed examination no modified inclusions of any kind were found inside the eutectic carbide globules. Also, calculations of the misfit between the crystal lattices of the hexagonal *Cr*_7_*C*_3_ carbide and all the inclusions that were found inside the REE-modified samples show that heterogeneous nucleation is unlikely to happen on modified inclusions. This is the reason why we decided to propose a new theoretical explanation for the modification of the eutectic carbide morphology that explains the phenomenon on the basis of first-principles thermodynamic calculations in terms of the phase-field modeling of the eutectic reaction^16,17^. A number of papers were published recently about the phase-field modeling of the eutectic reaction during steel solidification^18–22^. However, we used the novel principle first published by Ghosh^23^ to model the eutectic reaction in AISI D2 tool steel. The phase-field model includes the decomposition of the phase-boundary surface-energy term into the isotropic and anisotropic parts. A similar approach was reported for the phase-field modeling of spinodal decomposition^24^ and eutectic reaction modeling in the Ag-Cu system^25^. This principle turns out to be essential for describing the transition from a lamellar to a globular eutectic carbide morphology as a result of REE modification.

## Results

### Experimental

**Figure 1:**
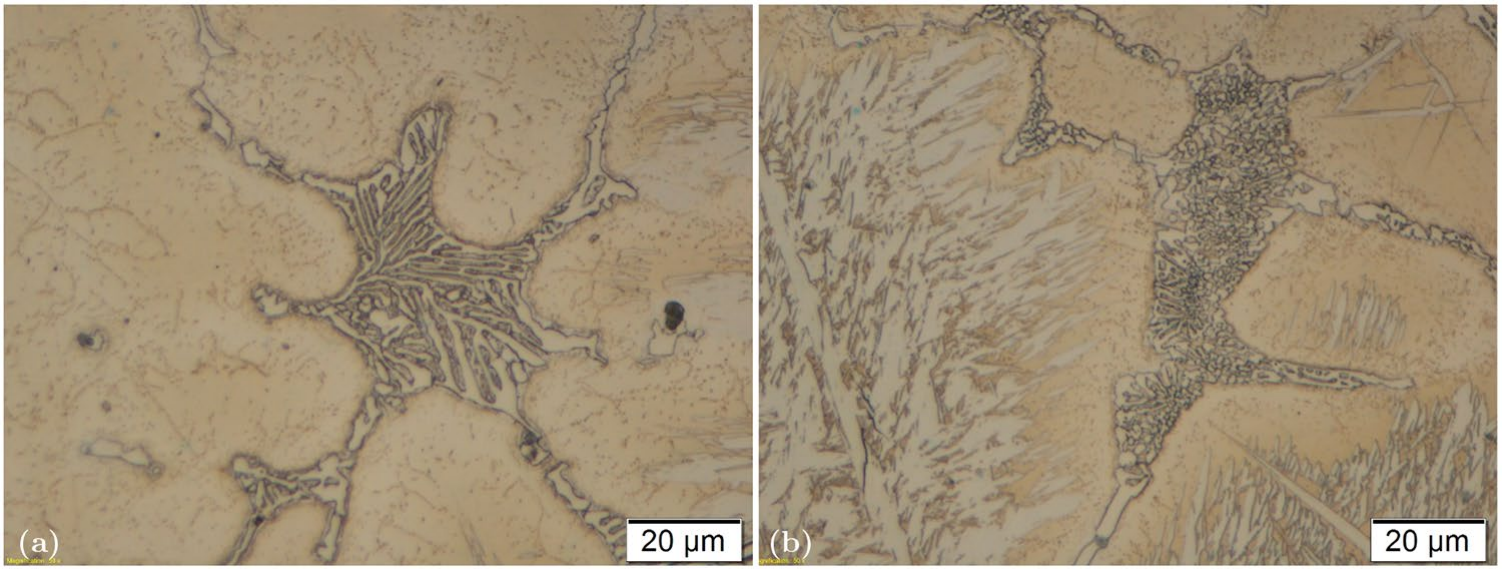
Comparison between morphology of eutectic carbide in (**a**) non-modified as-cast AISI D2 tool steel and (**b**) RE modified as-cast AISI D2 tool steel. (Optical microscopy image).

We modified the as-cast microstructure of the AISI D2 tool steel using REEs so as to change the morphology of the eutectic *Cr*_7_*C*_3_ carbides. The differences in the morphologies of the eutectic carbides in the non-modified and REE-modified samples can be seen from optical and SEM micrographs. Figure 1 shows the optical micrographs of the eutectic carbide in the (a) non-modified and (b) REE-modified samples.

**Figure 2:**
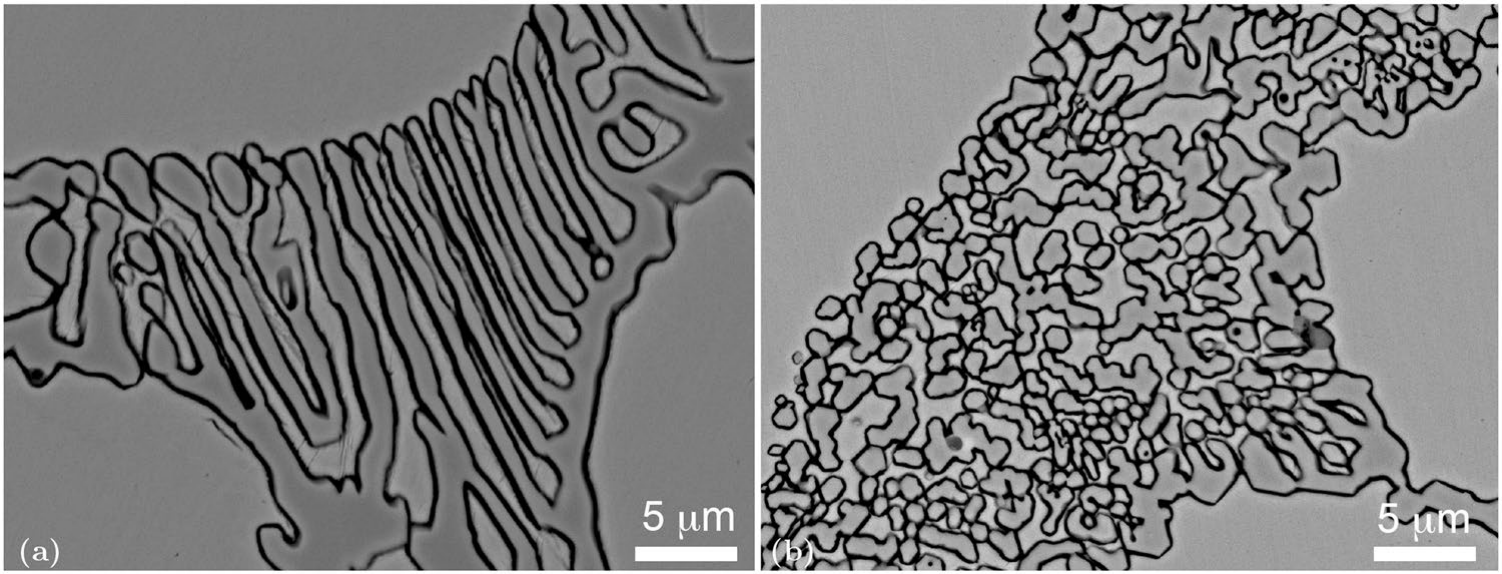
Comparison between morphology of eutectic carbide in (**a**) non-modified as-cast AISI D2 tool steel and (**b**) RE modified as-cast AISI D2 tool steel. (Backscattered electron images using Scanning electron microscopy).

The differences in the morphologies of the eutectic carbides are clearly seen in this figure as the carbide in the REE-modified sample is much finer. The carbide consists of small individual parts with an approximately globular shape that are surrounded by a matrix. The carbide in the non-modified sample has the more common shape of a lamellar eutectic. It consists of groups of parallel carbide lamellae. The same difference can be seen even more clearly in SEM micrographs based on backscattered-electron imaging (BEI) (Fig. 2). From this image it is also clear that globules of the eutectic carbide in the modified sample are actually hexagonally shaped, which is consistent with the fact that the *Cr*_7_*C*_3_ carbide crystal lattice is hexagonal.

A large number of eutectic carbide micrographs were taken on modified and non-modified samples at many different sites. All the micrographs were analyzed using image-recognition software that evaluates the anisotropy of surface features. The elongation of the eutectic carbides in the non-modified samples was, on average, three times greater than the elongation of the eutectic carbides in the REE-modified samples. The corresponding experimental data and an explanation of the method used were published in a separate article and can be found in reference^26^.

The addition of REEs to AISI D2 steel leads to the appearance of REE-containing non-metallic inclusions. The number of inclusions per *mm*^2^ is proportional to the amount of added REEs and is 70, 100, 120 for ingots #3, #4, #5, respectively. Nevertheless, the average size of the inclusions remains nearly the same in all three ingots.

### Modeling

The eutectic reaction was modeled using phase-field theory. Since three different phases are simultaneously present in the system, two order parameters were used in the phase-field model: the crystallinity and the non-dimensional concentration of the carbon. The commonly used shape of the total free energy^22^ was used in our model with an additional term that describes the anisotropic part of the surface free energy of the phase boundaries between the austenite and the *Cr*_7_*C*_3_ carbide (Equation 4). The time evolution of the system was calculated by solving the dynamic equation system (Equations 5 and 6) in two dimensions on a 4 *μm* × 4 *μm* domain with an 80 *nm* spatial resolution. The temperature of the system was controlled during the simulation, being linearly decreased over time until the phase transition was achieved. Then the temperature was maintained and the phase transition occurred at constant temperature. The simulation included thermal fluctuations in both order parameters that acted as homogeneous nuclei in the system. Heterogeneous nuclei were also included in the simulation as non-metallic inclusions of different types. Details of the modeling are described in Methods.

**Figure 3:**
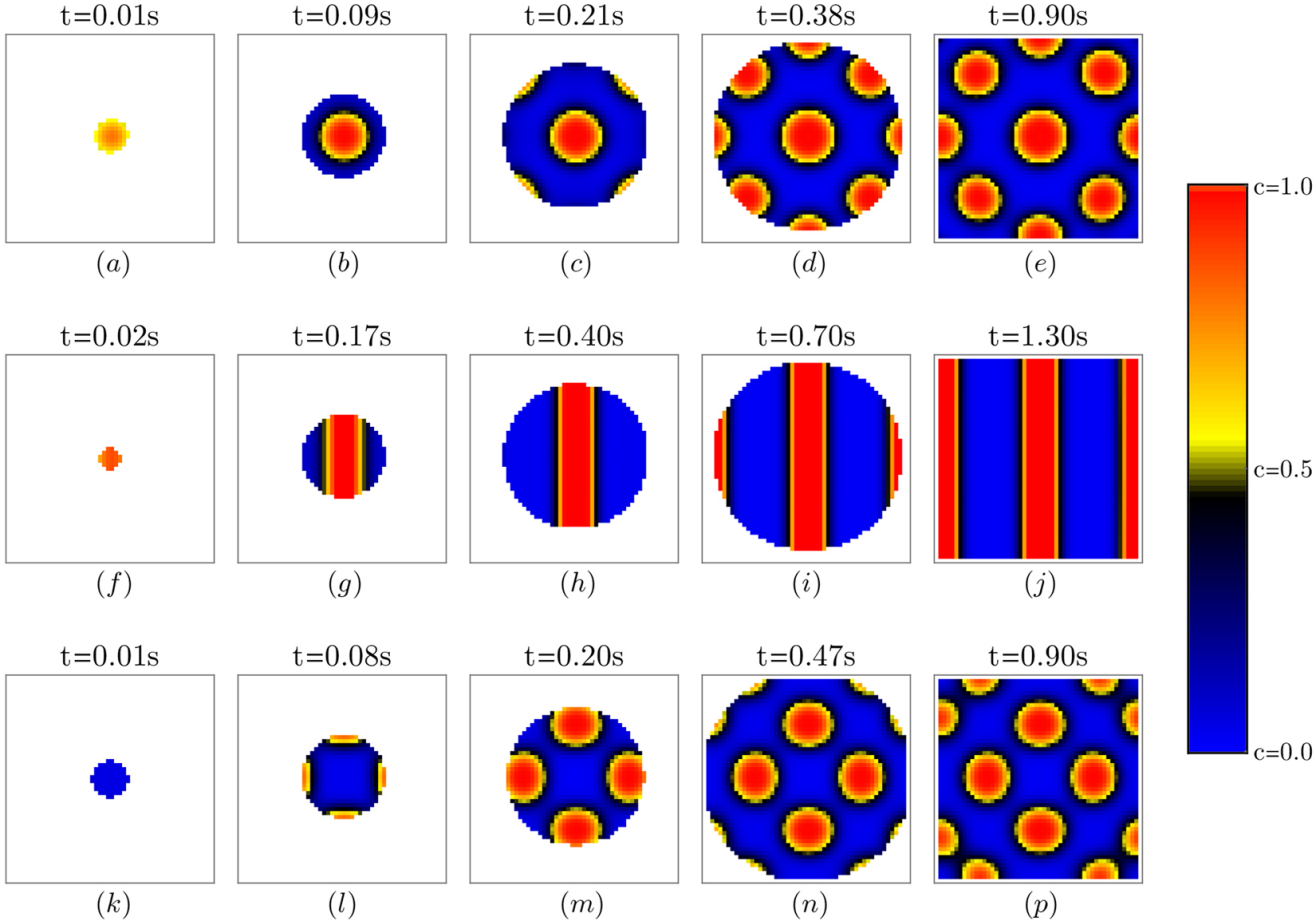
The results of the simulation for the three different solidification cases. The time evolution goes from left to right. Each row of the figure represents the separate solidification case. White color on the images represent the liquid phase (melt). Value of phase field order parameter *c* is plotted in colors represented in legend on the right. Red color represent *Cr*_7_*C*_3_ carbide and blue color represent austenite. The scale of the simulated system is 4 *μm* × 4 *μm*. Firs column (figures from (**a**–**e**)) represent the time evolution of the system where the ratio *B*(**n**_**x**_)/*κ*_*c*_ = 2000. Nucleation site for the reaction to begin was crystallographicaly more compatible with carbide than austenite. Second column (figures from (**f**–**j**)) shows the results for *B*(**n**_**x**_)/*κ*_*c*_ = 3000. Nucleation site again has lower crystal lattice misfits with carbide than austenite. Third column (figures from (**k**–**p**)) show the system time evolution where *B*(**n**_**x**_)/*κ*_*c*_ = 2000. In this case the nucleation site responsible for the initiation of the reaction shows lower misfit with the austenite crystal lattice in comparison to carbide.

The results of the simulation are presented as 50 × 50 pixel images of the calculation domain of the 4 *μm* × 4 *μm* size. Calculated two-dimensional phase fields for both order parameters were plotted on the domain in different colors (white - melt, red - *Cr*_7_*C*_3_ carbide, blue - austenite). The results for both the REE-modified and non-modified material solidification were simulated for two different types of heterogeneous nucleation sites that initiate the solidification. The first type of nucleation sites used in the simulation exhibits a smaller crystal-lattice misfit with the *Cr*_7_*C*_3_ hexagonal lattice than the austenite FCC crystal lattice and the second type was the opposite. The results for three different sets of parameters are presented in Fig. 3.

## Discussion

Two important facts can be taken from the comparison of the experimental and modeled results. The first is that the type of non-metallic inclusions in the melt does not influence the morphology of the eutectic carbides. It is generally understood that non-metallic inclusions modified by REEs represent good nucleation sites for the eutectic carbide and thus carbides with a modified morphology evolve in the REE-modified steel^14,15^. We found this to be highly unlikely, since during a detailed examination of the SEM BEI images of the modified samples no inclusions of any type were found inside the *Cr*_7_*C*_3_ carbides. Also, we calculated the misfits for all the lowest-index crystal faces of all the inclusions that were found on the EDS mapping with the crystal lattice of the *Cr*_7_*C*_3_ carbide. None of the inclusions showed a misfit small enough to represent an efficient nucleation site for the carbide. The simulations that we made clearly show that only one nucleation site is needed for the whole region of the eutectic to solidify in the decomposed final state. A single nucleation site is sufficient for the number of lamellae or globules to grow. In Fig. 3 we can see that one nucleation site resulted in nine globules (Fig. 3(e)), three lamellae (Fig. 3(j)) or twelve globules (Fig. 3(p)). In addition, just one nucleation site is needed for the numerous separated carbides to form. Numerous individual carbides can be formed from a nucleation site that does not even exhibit compatibility of the crystal lattice with the carbide (high misfits). Figure 3(k–p) show the time evolution of the solidification that started on the nucleation site compatible with an austenite FCC crystal lattice and nevertheless twelve globular eutectic carbides grew on the 4 *μm* × 4 *μm* domain. That clearly shows that the globularisation of the eutectic carbides has a different origin.

We are also in a position to explain the changed morphology of the eutectic carbides after the REE modification of the AISI D2 tool steel. The reason for the different morphologies of the eutectic carbides is the anisotropy of the surface energy of the phase boundary between the *Cr*_7_*C*_3_ carbide and the austenite. Large anisotropies result in a lammelar structure, since the carbide growth is energetically preferable in the directions parallel to the phase-boundary orientation with the lowest free energy. In the case of the globular eutectic there is no anisotropy of the phase-boundary surface energy and thus the carbide growth velocity is equal in all directions. The result is globular carbides.

The magnitude of anisotropic part of surface energy (*B*(***n***) in equation 4) is the parameter that governs the directions of the eutectic carbide growth. The high anisotropy of *B*(***n***) results in a lamellar carbide morphology, while a low anisotropy of *B*(***n***) leads to globular eutectic growth. *B*(***n***) can be calculated from the crystallographic properties of the carbide and the austenite. From the SEM micrographs and the crystallographic calculations we determined that the hexagonally structured chromium carbide and FCC austenite crystals meet with faces $\{11\bar{2}0\}$ (hexagonal lattice) and {730} (FCC lattice). The function *B*(***n***) in this case shows a high anisotropy, since all possible perpendicular phase boundaries exhibit much larger misfits. This results in a lamellar eutectic growth. After the modification the hexagonal carbide and the FCC austenite are stacked with $\{1\bar{1}00\}$ and {941} planes. This stacking has only a slightly higher strain energy than $\{11\bar{2}0\}$ to {730}, but it has a special property, i.e., when rotated by 120° we obtain the same boundary with the same misfit. For the hexagonal symmetry of the chromium carbide this means an equal growth rate in even the lowest crystallographic direction and, consequently, carbides in the shape of hexagonal globules. The transition from one regime to another due to REE modification was investigated by varying the different parameters in the model. It was found that the ratio between the isotropic and anisotropic parts of the surface energy is the crucial parameter that dictates the realization of two different regimes, i.e., the globular and the lamellar.

In the first regime (before modification) the contribution of the isotropic part (*κ*_*c*_) to the surface energy is small in comparison to the anisotropic part (*B*(***n***_*x*_)). The phase boundaries with the smallest possible misfit are realized, which minimizes the total free energy, even though the ratio between the areas of the phase-boundary surfaces versus the volume of carbide is large (lamellae). In the second regime (after modification) the isotropic part becomes non-negligible in comparison to the anisotropic part. The isotropic contribution to the surface energy favors the evolution of globules (spheres), since this morphology shows the lowest carbide area-to-volume ratio and consequently the smallest contribution of the isotropic surface energy. The compromise is made in the anisotropic part where the phase boundaries do not have the smallest possible misfit, but exhibit the lowest possible anisotropy in order to enable the globular growth and in this way minimize the total free energy.

We assume that the change in the ratio between the isotropic and anisotropic parts of the surface energy is a direct consequence of the REE addition. REEs have a very strong affinity for oxygen and sulphur. After the addition of the REE to the melt, complex oxysulphide non-metallic inclusions (*RE*_2_*O*_2_*S*) occur^15^ that tend to deplete the melt of sulphur and oxygen. These two elements are known to segregate to the grain boundaries during the solidification due to the low solid solubility of austenite^27–29^. Since the casting was conducted in an open induction furnace the levels of oxygen and sulphur in our material are high. In the case of the non-modified material large amounts of those two elements can be found at the grain boundaries, including the boundaries between the carbides and the austenite. In the case of the REE-modified steel the oxygen and sulphur segregate to the grain boundaries to a much lower extent since most of oxygen and sulphur are found in non-metallic inclusions. The amount of oxygen and sulphur that segregate to the boundaries between the carbide and the austenite changes the surface energy of such a boundary^30^. The coherency of the phase boundary is reduced due to the segregations and the number of dislocations is increased^31,32^. The anisotropic part of the surface energy is increased in comparison to the isotropic part when segregations are present. This is the reason why the ratio between the isotropic and anisotropic parts of surface energy of the phase boundary between the carbide and austenite differs between the REE-modified and non-modified material.

Quantitatively, this is shown in the simulation presented in Fig. 3. The reason for the change in the carbide morphology was the altered ratio between *κ*_*c*_ and the parameter *B*(***n***_*x*_). The function *B*(***n***) was simplified in our model so that the hexagonal anisotropy was approximated by isotropy, due to the long computational times. The results of the model therefore show spherically shaped carbides instead of hexagonal types in the case of REE-modified steel, but in comparison to the lamellae they prove the concept. It turns out that a relatively sharp transition from the lamellar to the globular regime occurs at the value of the non-dimensional expression $\frac{B({{\boldsymbol{n}}}_{x})}{{\kappa }_{c}}=2500$. In Fig. 3 we can see that the value of $\frac{B({{\boldsymbol{n}}}_{x})}{{\kappa }_{c}}=2000$ leads to a globular eutectic, while the value of 3000 leads to a lammelar eutectic. This proves that the oxygen and sulphur contents, control by REE additions, can lead to the transition from a lammelar to a globular eutectic in AISI D2 tools steel.

## Methods

### Sample preparation

20 kg of AISI D2 tool steel was remelted in the induction furnace. It was cast at 1600°C into 5 cylindrical metallic molds. Before casting the one fifth of the melt in to the first mold, aluminum was added to the melt to de-oxidize it. After the first casting Ca-Si (70% Si, 30% Ca) was added to the remaining melt to additionally de-oxidize and de-sulphurize it. The second casting was made after the Ca-Si treatment. Before the third casting the first amount of REEs was added to the remaining melt in the form of mischmetal so as to modify the microstructure. The composition of the Ce mischmetal is presented in Table 1. The same procedure was repeated for the fourth and fifth castings. The procedures for every ingot treatment are presented in Table 2. In this way we obtained five 4-kg ingots of the same shape and size, but with slightly different chemical compositions, which were determined by chemical analyses. The results are presented in Table 3.

**Table 1:** Chemical composition of Ce mischmetal in weight percent.

Element	Ce	La	Nd	Pr
Wt.%	45–55	20–35	15–20	5–8

**Table 2:** Ingot chemical composition was modified by small amounts of additions.

Ingot Number	#1	#2	#3	#4	#5
Additions	Al	Al, Ca-Si	Al, Ca-Si, Ce Mischmetal	Al, Ca-Si, Ce Mischmetal	Al, Ca-Si, Ce Mischmetal

**Table 3:** Chemical composition of five ingots.

Element	C	Si	Mn	Cr	Cu	Ni	Mo	V	Ce	La	Nd	Pr	Fe
Ingot #1	1,43	0,19	0,21	10,4	0,08	0,17	0,60	0,83	0,00	0,00	0,00	0,00	bal.
Ingot #2	1,42	0,23	0,21	10,1	0,08	0,17	0,60	0,75	0,00	0,00	0,00	0,00	bal.
Ingot #3	1,41	0,23	0,22	9,8	0,08	0,18	0,59	0,70	0,007	0,004	0,004	<0,002	bal.
Ingot #4	1,43	0,22	0,22	10,2	0,08	0,17	0,62	0,78	0,011	0,006	0,004	<0,002	bal.
Ingot #5	1,41	0,22	0,21	10,3	0,08	0,17	0,59	0,80	0,013	0,008	0,005	<0,002	bal.

Ingots number 3, 4 and 5 contain different amount of rare earth elements.

After solidification the samples were cut from the geometrical center of the ingots using a water-jet slicer. The samples were subsequently prepared for optical and electron microscopy using the standard metallographic procedures of polishing and etching with Nital (*C*_2_*H*_6_*O* and *HNO*_3_). The largest differences between the morphologies of the eutectic carbides were observed when we compared the non-modified samples (ingot #1 and #2) with the samples containing the largest weight concentration of REEs (ingot #5). All the comparisons of the microstructures presented in the Results section are between samples from ingot #2 (0 wt.% of RE) and ingot #5 (0.027 wt.% of RE).

### Characterisation

The as-cast microstructure of the non-modified and modified AISI D2 tool steel samples were analyzed using optical and scanning electron microscopy. Optical microscopy was performed on a Microphot FXA, Nikon microscope. The scanning electron microscopy and the microanalyses involved a Jeol JSM - 6500F scanning electron microscope. The micrographs were further analyzed with a quantitative metallographic image-processing tool that determines the anisotropy of the microstructural features. The analytical tool was developed in our laboratory and programed in open source GNU Octave programing environment^26^.

### Modeling and Calculations

The phase-field model, based on the Landau theory of phase transitions, was used to simulate the measured phenomenon. Two phase-field order parameters were used to model the solidification from the liquid phase (melt) to two separated solid phases (*Cr*_7_*C*_3_ carbide and austenite). The first-order parameter, i.e., the crystalinity (*ρ*), indicates whether part of the system is in the liquid or solid state (negative and positive values of *ρ*). The second-order parameter (*c*) describes the decomposition of the solid parts of system into two separated phases and can be interpreted as a non-dimensional carbon concentration. Minimising the free energy function with respect to both order parameters gives us the equilibrium states of the system for a given set of parameters and the time evolution of the system can be calculated. Different phenomenological free-energy density forms are used for eutectic reaction modeling. A polynomial or logarithmic shape of the free-energy density is the usual choice since we need two double-well potentials that are coupled via a temperature term. For the free-energy density *ρ* dependency we chose the polynomial form and for the *c* dependency of the free-energy density we used a logarithmic form. In this way we described the coupled system of solidification of the liquid phase and the decomposition of two solid phases. The free-energy density in non-dimensional form that we used in the model reads:

1 $$\begin{array}{rcl}f(\rho ,c) & = & \frac{a}{4}{\rho }^{4}-\frac{|r|}{2}{\rho }^{2}+[\alpha {\rm{\Delta }}T-{\rm{\Omega }}(c(1-c)+{c}_{L}(1-{c}_{L}))]\,\rho \\  &  & +\beta \,({cln}\,({c})+(1-{c}){ln}(1-{c}))\end{array}$$

*a*, *r*, *α*, *β* and Ω are parameters of the model and were determined by different characteristics of the system that were obtained by experiment. Δ*T* represents the difference between the absolute temperature of the system (*T*) and temperature of the phase transition (*T*_*E*_), which is the eutectic temperature Δ*T* = *T* − *T*_*E*_. *c*_*L*_ represents the value of the order parameter *c* in the liquid phase. Together with the surface energy of the phase boundaries (*f*_*S*_) we can calculate the total free energy that will be minimized in order to obtain the time evolution of the system dynamics. The total free energy (*F*) can be written as the volume integral of all the contributions to the free-energy density.

2 $$F(\rho ,c)={\int }_{V}\,(f(\rho ,c)+{f}_{S}(\rho ,c))dV$$

The surface energy of the phase-boundaries contribution usually has the shape of a gradient penalty term^33^ ${f}_{S}(\rho ,c)={\kappa }_{\rho }\frac{1}{2}{(\nabla \rho )}^{2}+{\kappa }_{c}\frac{1}{2}{(\nabla c)}^{2}$. The first term represents the free-energy density contribution of the phase boundary between the liquid and the solid and the second term describes the free-energy density of the phase boundaries between two solid phases. $\nabla$ represents the gradient operator. *κ*_*ρ*_ and *κ*_*c*_ are the gradient penalty coefficients and represent the magnitudes of the gradient-penalty terms. Since the orientation of the crystal lattices of both phases that form the phase boundary affects the phase-boundary free energy, *κ*_*c*_ is in general a tensor. In this way the gradient penalty’s contribution to the total free-energy dependence on the orientation of the phase boundary is described. We decided to decompose the gradient-penalty term ${\kappa }_{c}\frac{1}{2}{(\nabla c)}^{2}$ into two separate terms, each describing the separate phenomena that contribute to the free surface energy of the phase boundary between two solid crystalline phases. The first term describes the isotropic free-energy contribution of the phase boundary. This can be described by the standard gradient penalty term with the scalar gradient-penalty coefficient, since this part of the surface free energy does not depend on the orientation of the phase boundary. The second term describes the lattice strain energy at the boundary between two crystalline phases that occurs due to the misfit between two crystal lattices that meet at the phase boundary. This term is anisotropic and can be derived from the theory of elasticity. The phase-boundary free-energy density can be written as

3 $${f}_{S}(\rho ,c)={\kappa }_{\rho }\frac{1}{2}{(\nabla \rho )}^{2}+{\kappa }_{c}\frac{1}{2}{(\nabla c)}^{2}+\frac{1}{2}{C}_{ijkl}{\varepsilon }_{ij}{\varepsilon }_{kl}$$

where *κ*_*c*_ now represent the scalar coefficient of the chemical contribution to the phase-boundary free-energy density between two solid phases. *C*_*ijkl*_ represents the stiffness tensor and *ε*_*ij*_ is the strain tensor. This expression can be further simplified using the approximation first derived by Cahn^33^: $\frac{1}{2}{C}_{ijkl}{\varepsilon }_{ij}{\varepsilon }_{kl}\approx B({\boldsymbol{n}})\,{(c-\bar{c})}^{2}$. ***n*** is the unit vector normal to the phase boundary and *B*(***n***) is the function that describes the strain free-energy contribution’s dependency on the phase-boundary orientation. A full formulation of the strain term’s approximation can be found in the reference^24^. *B*(***n***) values were calculated from the austenite FCC and *Cr*_7_*C*_3_ hexagonal crystal lattice misfits for different orientations. The total free energy can be written as

4 $$\begin{array}{rcl}F(\rho ,c) & = & {\int }_{V}\,[\frac{a}{4}{\rho }^{4}-\frac{|r|}{2}{\rho }^{2}+[\alpha {\rm{\Delta }}T-{\rm{\Omega }}(c(1-c)+{c}_{L}(1-{c}_{L}))]\rho \\  &  & +\,\beta \,({cln}({c})+(1-{c})\,{ln}\,(1-{c}))+{\kappa }_{\rho }\frac{1}{2}{(\nabla \rho )}^{2}\\  &  & +\,{\kappa }_{c}\frac{1}{2}{(\nabla c)}^{2}+B({\boldsymbol{n}})\,{(c-\bar{c})}^{2}]\,dV\end{array}$$

The differentiation of *F* by both order parameters gives us dynamic equations of the system^22^.

5 $$\frac{\partial \rho }{\partial t}={M}_{\rho }\frac{\delta F}{\delta \rho }+{\eta }_{\rho }$$

6 $$\frac{\partial c}{\partial t}={\nabla }^{2}\,({M}_{c}\frac{\delta F}{\delta c})+{\eta }_{c}$$

*M*_*ρ*_ and *M*_*c*_ are mobility of the species in the liquid phase and solid mobility of carbon. Terms *η*_*ρ*_ and *η*_*c*_ are Langevine noise functions that describe the thermal fluctuations of the order parameters^17^. *η*_*ρ*_ and *η*_*c*_ are defined so that at a random time at a random site of the system order parameter is perturbed for the Gaussian distributed value.

Equations 5 and 6 represent the non linear forth order system of partial differential equations. The solution of the equations represents the time evolution of the system for the given parameters. Equations were numerically solved in two dimensions for periodic boundary conditions. Discretization on the two dimensional mesh of the size 50 × 50 was made by finite volume method, to obtain the system of 5000 non linear algebraic equations. the system was solved by Newton-Raphson method as described in Numerical Recipes in C^34^. The source code for simulation was coded in C programing language. The results were obtained in the form of matrices that represented the spatial distribution of both order parameters for each time step. Matrices were plotted to figures by open source GNU Octave program.

During the simulation the parameter Δ*T* was varied in order to simulate the cooling of the melt. The constant heat flow out of the domain results in a linearly decreasing temperature until the phase transition occurs. At the phase transition the latent heat is released and the temperature was maintained so that whole reaction occurred at a constant temperature. The temperature at which the phase transition occurred was between −10 *K* > Δ*T* > −15 *K*. The degree of under cooling depends on the randomly positioned nucleation sites of random size and random type that were implemented in the model in the same way as the thermal fluctuations. Two types of nucleation sites were used. The difference between the two was the compatibility of the crystal lattice of nucleation site with the crystal lattice of the austenite and the *Cr*_7_*C*_3_ carbide. The first type has a smaller crystal lattice misfit with the *Cr*_7_*C*_3_ carbide in comparison to the austenite and for second type the opposite situation applies.

## Conclusion

In our investigation we successfully modified AISI D2 tool steel with REEs so that the morphology of the eutectic carbides in the as-cast microstructure was changed. Furthermore, a phase-field model of eutectic reaction was proposed that offers an explanation for the observed phenomena on the basis of a thermodynamic first-principles calculation. From the combination of the experimental and modeling results we were able to draw two important conclusions. The first is that the modified non-metallic inclusions in REE-modified AISI D2 tool steel do not influence the morphology of the eutectic carbides. The second conclusion is that the decreased ratio between the isotropic and anisotropic parts of the phase-boundary surface free energy leads to a globular eutectic morphology instead of a lamellar type. The decrease in the ratio between the isotropic and anisotropic parts of the phase-boundary surface energy is a consequence of the altered amounts of oxygen and sulphur that segregate to the phase boundaries.
